# Superflexible Inorganic Ag_2_Te_0.6_S_0.4_ Fiber with High Thermoelectric Performance

**DOI:** 10.1002/advs.202207642

**Published:** 2023-03-08

**Authors:** Yanqing Fu, Shiliang Kang, Hao Gu, Linling Tan, Chengwei Gao, Zaijin Fang, Shixun Dai, Changgui Lin

**Affiliations:** ^1^ Laboratory of Infrared Materials and Devices The Research Institute of Advanced Technologies Ningbo University Ningbo 315211 P. R. China; ^2^ Key Laboratory of Photoelectric Detection Materials and Devices of Zhejiang Province Ningbo 315211 P. R. China; ^3^ Engineering Research Center for Advanced Infrared Photoelectric Materials and Devices of Zhejiang Province Ningbo 315211 P. R. China; ^4^ Guangdong Provincial Key Laboratory of Optical Fiber Sensing and Communications Institute of Photonics Technology Jinan University Guangzhou 511443 P. R. China

**Keywords:** ductile inorganic semiconductors, flexible fibers, thermoelectrics, wearable electronics

## Abstract

Fiber‐based inorganic thermoelectric (TE) devices, owing to the small size, light‐weight, flexibility, and high TE performance, are promising for applications in flexible thermoelectrics. Unfortunately, current inorganic TE fibers are strictly constrained by limited mechanical freedom because of the undesirable tensile strain, typically limited to a value of 1.5%, posing a strong obstacle for further application in large‐scale wearable systems. Here, a superflexible Ag_2_Te_0.6_S_0.4_ inorganic TE fiber is demonstrated that provides a record tensile strain of 21.2%, such that it enables various complex deformations. Importantly, the TE performance of the fiber shows high stability after ≈1000 cycles of bending and releasing processes with a small bending radius of 5 mm. This allows for the integration of the inorganic TE fiber into 3D wearable fabric, yielding a normalized power density of 0.4 µW m^−1^ K^−2^ under the temperature difference of 20 K, which is approaching the high‐performance Bi_2_Te_3_‐based inorganic TE fabric and is nearly two orders of magnitude higher than the organic TE fabrics. These results highlight that the inorganic TE fiber with both superior shape‐conformable ability and high TE performance may find potential applications in wearable electronics.

## Graphical abstract

Inorganic Ag_2_Te_0.6_S_0.4_ fiber with both superb flexibility and high thermoelectric performance is successfully fabricated by a molten core approach. The resulting Ag_2_Te_0.6_S_0.4_ fiber provides a record tensile strain of 21.2%, such that it enables various complex deformations. The 3D wearable fabric made from Ag_2_Te_0.6_S_0.4_ fiber demonstrates its potential for flexible thermoelectric devices.

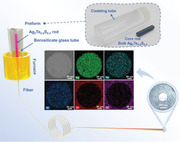

## 1 Introduction

Flexible thermoelectric (TE) generators are receiving increasing attention in wearable electronics because of their small volume, no moving parts, and high reliability.^[^ ^1^, ^2^, ^3^ ^]^ Fiber‐based TE devices have the unique structure feature of large length‐to‐diameter ratio and natural flexibility and can be woven and wound through various directions, making them attractive in large‐scale wearable systems.^[^ ^4^, ^5^ ^]^ A 3D TE device prepared by weaving with TE fibers can efficiently convert heat into electricity by using the temperature difference between the human body and ambience in a vertical direction.^[^ ^6^, ^7^ ^]^ Recently, a variety of novel TE fibers have been fabricated based on different strategies, such as p‐n segmented TE fibers fabricate by an alternate wet‐spinning method,^[^ ^8^ ^]^ formation of p‐type and n‐type segments by coating yarns with TE materials,^[^ ^9^, ^10^ ^]^ gelation extrusion strategy to prepare continuous p/n TE fibers,^[^ ^11^ ^]^ and develop a *π*‐type TE fiber using oleamine doping combined with electrospray technology.^[^ ^7^ ^]^ However, despite intensive research efforts and advances in fabrication of TE fibers, it has been challenging to prepare TE devices with both excellent flexibility and high TE performance.

The current and primary fiber‐based TE devices consist of inorganic, organic, or inorganic/organic hybrid fibers.^[^ ^12^, ^13^ ^]^ The state‐of‐the‐art inorganic TE fibers (e.g., Bi_2_Te_3_, SnSe, and PbTe fibers) show super‐high TE performance because of their narrow band gap, low thermal conductivity, and unique structure.^[^ ^14^, ^15^ ^]^ For instance, p‐type Bi_0.5_Sb_1.5_Te_3_ and n‐type Bi_2_Se_3_ fibers were fabricated using thermal drawing method. These TE fibers were then assembled into textile to construct TE fabric, generating an output voltage of 5 mV at a Δ*T* of 8 K.^[^ ^16^ ^]^ Similar works were reported in Bi_2_Te_3_‐based fibers. The as‐fabricated fibers possess a high power factor of 1.32 mW m^−1^K^−2^, and the TE cup generator covered with Bi_2_Te_3_ TE fibers reaches a power of 0.47 mW under ΔT ≈19 K.^[^ ^17^ ^]^ However, the intrinsic rigidity and brittleness of these inorganic TE materials induce the low tensile strain of inorganic TE fibers, on the order of 0.3% to 1.5%, leading to very limited mechanical freedom.^[^ ^16^, ^17^ ^]^ This poses a strong obstacle to attach them to curved surfaces with arbitrary geometry and thus limits their further application in large‐scale wearable platforms. In contrast, organic TE fibers exhibit unique flexibility, low density, and ease of fabrication.^[^ ^18^, ^19^ ^]^ PEDOT: PSS TE fiber is commonly fabricated by wet‐spinning method, which directly injects the PEDOT: PSS‐based formulation into a coagulation bath of H_2_SO_4_, and the as‐obtained fibers exhibited a power factor of 30 µW m^−1^ K^−2^.^[^ ^20^ ^]^ The TE performance can be further improved by introducing a drawing step in the wet‐spinning process, resulting in a power factor of up to ≈115 µW m^−1^ K^−2^.^[^ ^21^ ^]^ While the poor electrical transport property and low power factor still limit their potential in high‐performance wearable devices. By combining the inorganic fillers into the organic host, hybrid TE fibers normally show superior TE performance and increased flexibility. To illustrate, Ag_2_Te nanocrystals can coat on the surface of nylon fibers to form a flexible composite TE fiber. At the ΔT of 20 K, a TE generator prepared with such fiber can generate the power of about 0.8 nW.^[^ ^22^ ^]^ In addition to simply coating inorganic materials on organic substrates, other routes to rationally mix the organic and inorganic fibers are also explored. PEDOT: PSS/Te‐nanowire composite fibers were fabricated by the typical solution method, showing an optimized power factor of 100 µW m^−1^ K^−2^, which was nearly five orders of magnitude higher than that of pure PEDOT: PSS fiber.^[^ ^23^ ^]^ Nevertheless, the achievement of high‐quality inorganic/organic hybrid fibers remains a long‐standing goal owing to the inevitable aggregation and oxidation of inorganic fillers during the fabrication.^[^ ^24^, ^25^ ^]^ Research and development of new‐generation TE fibers with both promising TE properties and flexibilities are urgently needed to realize self‐power supply for wearable electronics.

The recently discovered ductile inorganic semiconductors based on silver sulfide realize the integration of high electrical mobility and mechanical flexibility in one single‐phased inorganic material,^[^ ^26^ ^]^ which opens a new utility in flexible inorganic TE materials.^[^ ^27^, ^28^, ^29^, ^30^ ^]^ For instance, a unique inorganic material Ag_2_Te_0.6_S_0.4_ with both extraordinary plastic deformability and high room temperature TE performance is developed.^[^ ^31^ ^]^ It can be reshaped into flexible slice without compromising the TE performance. Interestingly it exhibits glass‐like thermal transport behavior but shows electron transport property characteristic of a crystalline semiconductor, pushing the TE figure of merit (*zT*) to 0.2, an attractive value for flexible TE materials. However, the flexibility of the existing bulk or 2D planar configuration is restricted, whereas for various human‐interfaced devices arbitrary bending and deformation are constantly involved during use. In addition, it is difficult for the bulk or planar devices to integrate into fabrics in a large area to take full advantage of wearable devices.^[^ ^32^, ^33^ ^]^ To date, the fabrication of inorganic Ag_2_Te_0.6_S_0.4_ fibers and their potential in flexible TE devices are not yet to be reported.

In this work, we demonstrate a molten core approach that enables scalable fabrication of inorganic Ag_2_Te_0.6_S_0.4_ fiber with both superb flexibility and high TE performance for the first time. An extraordinary tensile strain of 21.2% is achieved, setting the record for inorganic TE fibers. The superior plastic deformability of the fiber originates from the formation and evolution of shear bands from its amorphous phase. After hundreds of bending and releasing cycles with a small bending radius of 5 mm, the fibers present uncompromised TE performance that has long been considered the key obstacle toward practical applications. A prototype of 3D wearable fabric constructed by Ag_2_Te_0.6_S_0.4_ fibers shows a normalized power density of 0.4 µW m^−1^ K^−2^ under the temperature difference of 20 K, which is approaching to the Bi_2_Te_3_‐based inorganic TE fabric and is about two orders of magnitude higher than the organic TE fabrics. The combination of excellent flexibility and high TE performance may greatly promote the applications of Ag_2_Te_0.6_S_0.4_ fibers in wearable electronics.

## 2 Results and Discussion

### 2.1 Thermal Drawing Fabrication of Superflexible Inorganic Ag_2_Te_0.6_S_0.4_ Fibers

**Figure 1:**
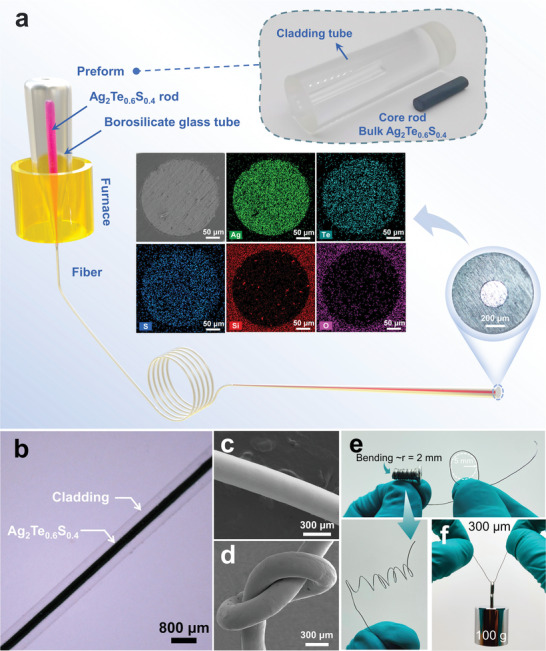
a) Schematic of the fiber drawing process. The insert shows the photographs of the fiber preform, cross‐section of the as‐drawn fiber and the SEM mapping of different elements in the Ag_2_Te_0.6_S_0.4_ fiber with the core diameter of 300 µm. b) View of the Ag_2_Te_0.6_S_0.4_ fiber. c) SEM images of Ag_2_Te_0.6_S_0.4_ fiber without cladding and d) a knotted fiber showing excellent flexibility. e) Photographs of the Ag_2_Te_0.6_S_0.4_ fiber without cladding coiling on a screw with a radius of 2 mm and f) being loaded with an object of 100 g.

Ag_2_Te_0.6_S_0.4_ materials exhibit superior flexibility and excellent TE performance (Figure S1, Supporting Information), which is promising to prepare fiber for flexible TE devices. However, due to the lack of softening temperature,^[^ ^31^, ^34^, ^35^ ^]^ the Ag_2_Te_0.6_S_0.4_ fibers cannot be prepared through direct preform‐drawing method, which is normally performed near the softening point. Alternatively, the molten core method has been proved to be an expansive and straight‐forward fiber‐drawing approach that enables integration of various functional materials including glasses, metals, polymers or semiconductors into flexible fiber, where the core and the cladding are, respectively, made of a low and high melting‐point materials.^[^ ^36^, ^37^ ^]^ In this molten core process, the core is in the molten state while the cladding, which is treated as a container and co‐drawn with the core, is just softened for fiber‐drawing. As shown in **Figure** **1**a, Ag_2_Te_0.6_S_0.4_ sample with its melting temperature of around 1040 K is selected as the core and a borosilicate glass with its drawing temperature of 1100 K is used as the cladding. The cylindrical core rod with the size of Φ6.0 × 50.0 mm is inserted into the cladding tube with an inner diameter of 6.1 mm and an external diameter of 20.0 mm to form a preform. The preform is fed into a fiber‐drawing tower under a controlled feeding speed and then fiber‐drawing is performed at a temperature of 1100 K. At this temperature, the core is a fluid melt, while the cladding starts to soften and then be drawn into fibers at a certain drawing speed. During the fiber‐drawing process, the fiber core diameter is distributed within 100–700 µm, depending on the fiber‐drawing speed and preform feed speed. It is worthy to mention that this technique is scalable to obtain long fiber (with a diameter of 300 µm and length of ≈20 m in this work) from individual production run.^[^ ^37^ ^]^

The insert of Figure 1a presents the image of the fiber cross section with classic core‐cladding configuration, where the core and cladding diameters are around 300 and 950 µm, which coincides well with the original core‐cladding diameter ratio of the preform. The scanning electron microscopy (SEM) image and the corresponding energy‐dispersive spectra (Figure 1a) of the TE fiber clearly indicate that the constituent elements of the functional TE core are homogenously distributed in the core region of the fiber. Figure 1b shows that the fiber exhibits good core continuity and a clear interface between the concentric glass cladding and Ag_2_Te_0.6_S_0.4_ core. The SEM image (Figure 1c) of a bare TE fiber without cladding shows that the surface of the fiber is highly smooth. We also note that the resulting TE fiber without cladding exhibits extraordinary room‐temperature flexibility so that it can be tied into a knot without obvious damage in structure (Figure 1d and Movie S1, Supporting Information). Additionally, it can stay intact after coiling on a screw with a radius of 2 mm and can lift a weight of 100 g without crack (Figure 1e). The superior robustness and flexibility of the TE fiber are believed to be critical for body conformation, comfort and aesthetics when applied in large‐scale human‐interfaced wearable devices.

### 2.2 TE Performance Characteristics of Ag_2_Te_0.6_S_0.4_ Fibers

**Figure 2:**
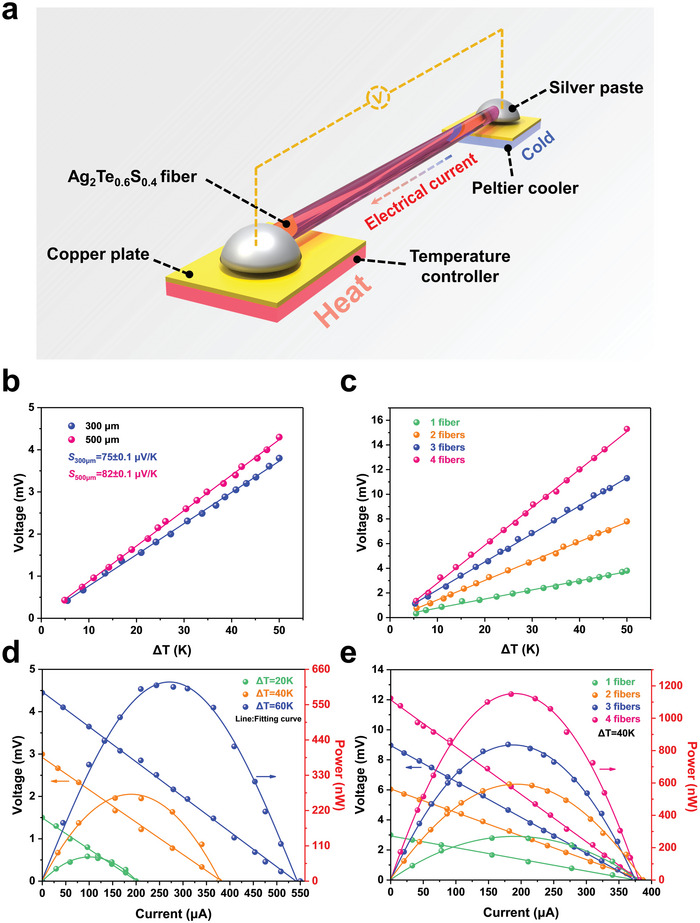
a) Schematic illustration for measuring thermoelectric properties of fibers. b) Functional relationship between output voltage and temperature difference (Δ*T*) for Ag_2_Te_0.6_S_0.4_ fibers with different diameters, the lines with the same color are the corresponding fitting results. c) Voltage, current, and power output of a Ag_2_Te_0.6_S_0.4_ fiber with the diameter of 300 µm and length of 6 cm at various temperature differences. d) Output voltage of Ag_2_Te_0.6_S_0.4_ fiber as a function of the temperature difference with different number in series. e) Voltage, current, and power output of the Ag_2_Te_0.6_S_0.4_ fibers with different number in series at a temperature difference of 40 K.

Based on the Seebeck effect, we constructed an experimental apparatus, consisting of a heat source, a heat sink, and a segment of TE fiber, to study the TE performance of Ag_2_Te_0.6_S_0.4_ fiber, as presented in **Figure** **2**a. During the experiment, the two ends of the TE fiber were contacted with the heat source and the heat sink and fixed with silver paste. A constant temperature field was provided by a temperature controller at the hot end, while the cold end temperature was maintained at 300 K using a commercial Peltier module in contact with a circulating water cooler. To ensure quick and accurate transfer of the temperature to the TE fiber, two copper plates were introduced at both hot and cold ends. A digital multimeter (Keithley DM6500) was connected to the two ends of TE fiber to record real‐time dynamic data. Figure 2b shows the output voltage as a function of the temperature difference based on two single TE fibers with the diameters of 300 and 500 µm, respectively. The output voltage increases linearly with the temperature difference, yielding the slopes (Seebeck coefficient (*S*)) of 75 and 82 µV K^−1^. The output voltage can be further amplified by series electrical connection between the terminals of each fiber. As evidenced by Figure 2c, with the increase of the number of TE fiber, the output voltage approximately amplifies proportionally. Compared to the single TE fiber, the output voltage of the TE fiber composed of 4 segments of fibers increases from 3.8 to 15.3 mV at the same temperature difference of 40 K. By using the four‐probe method, the electrical resistivity of fiber with the length of 1.89 cm and the diameter of 300 µm was measured to be 0.886 mΩ cm, which is close to the p‐type Bi_1.5_Sb_0.5_Te_3_ TE fibers and seven order of magnitude lower than that of SWCNT/PVA TE fibers.^[^ ^12^, ^34^ ^]^ Such low electrical resistivity is conducive to obtaining high output power. Figure 2d shows the output voltage and power as a function of output current under various temperature differences for a single TE fiber. The maximum open‐circuit voltage and output power increase with the increase of temperature gradient, reaching 4.4 mV and 606 nW under the temperature difference of 60 K, respectively, and the corresponding load resistances were recorded in Figure S2a (Supporting Information). Owing to the higher Seebeck coefficient and electrical conductivity, the output power of a single Ag_2_Te_0.6_S_0.4_ fiber is about 5 times higher than that of PEDOT: PSS/tellurium nanowires composites TE fiber (45 nW, ΔT = 41 K), and four order of magnitude higher than that of SWCNT/PVA‐based fiber (2.5 pW, ΔT = 20 K).^[^ ^11^, ^38^ ^]^ The output performance (output voltage and power) can be further enhanced with increasing the number of fibers in series. As illustrated in Figure 2e, under the temperature gradient of 40 K, the power increases from 265 to 1154 nW as the number of fibers varies from 1 to 4, and the corresponding applied load resistance is 7.9–31.7 Ω (Figures S2b and S3, Supporting Information). This demonstration not only exhibits the superior TE performance of the fiber, but also shows the multiplication effect of the energy harvesting ability from single to multiple TE fibers.

### 2.3 Superior Flexibility of Ag_2_Te_0.6_S_0.4_ Fibers

**Figure 3:**
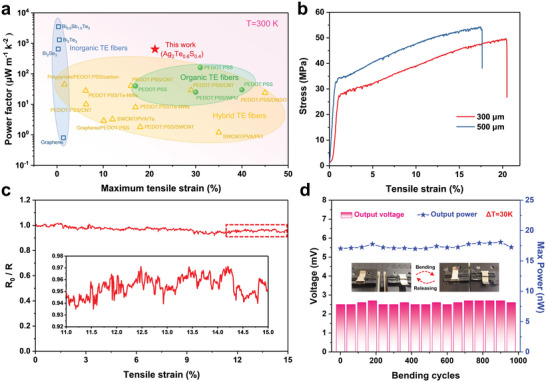
a) Maximum tensile strain with the corresponding power factor of the Ag_2_Te_0.6_S_0.4_ fiber is compared with those of organic and inorganic TE fibers in the literature. The detailed dated and the related references are listed in Table S1 (Supporting Information). b) Measured strain‐stress curve for tensile test of Ag_2_Te_0.6_S_0.4_ fibers with different diameters. c) The change of *R*_0_/*R* of Ag_2_Te_0.6_S_0.4_ fiber under different strains. The insert shows an enlarged view of the recorded data. d) The change of output voltage and maximum output power of Ag_2_Te_0.6_S_0.4_ fiber at a Δ*T* of 30 K after ≈1000 bending and releasing cycles with a radius of 5 mm.

Excellent flexibility is prerequisite for the implementation of fiber‐based wearable TE devices, which is required to fit small heat sources with complex curve shapes. Conventional TE fibers based on inorganic semiconductors such as Bi_2_Te_3_, Bi_0.5_Sb_1.5_Te_3_ and Bi_2_Se_3_ show poor mechanical flexibility (the tensile strain is typically limited to 1.5%)^[^ ^16^, ^17^ ^]^ because of their strong ionic or covalent bonds, severely limiting their potential applications in flexible wearable TE devices, as shown in **Figure** **3**a. Compared to these inorganic TE fibers, the prepared Ag_2_Te_0.6_S_0.4_ fiber without cladding exhibits exceptional flexibility with a maximum tensile strain of 21.2% (Figure 3b, and Movie S2, Supporting Information), which is comparable to some organic fibers and inorganic/organic hybrid fibers. In addition, the power factor of Ag_2_Te_0.6_S_0.4_ fiber is around 630 µW m^−1^ K^2^, which is comparable to the high‐performance Bi_2_Se_3_ inorganic TE fiber (650 µW m^−1^ K^2^), and almost one order of magnitude higher than the organic and hybrid TE fibers. Taken together, the Ag_2_Te_0.6_S_0.4_ TE fiber combines superior flexibility and high TE performance, showing significant promise in the fields of flexible TE devices for wearable energy harvesting.

We also test the service stability of Ag_2_Te_0.6_S_0.4_ fiber by monitoring the dynamic response of the fiber to resistance change (*R*_0_/*R*, where *R*_0_ represents the original resistance and *R* is the measured resistance under deformation) during the stretching process. As shown in Figure 3c, with increasing tensile strain, the electrical resistance of the fiber exhibits slight increase of 5% in the strain range from 0–15%. This resistance change of the fiber during stretching has little effect on the output voltage (Figure S4, Supporting Information). It is worth mentioning that the fiber also exhibits excellent fatigue resistance. The variance of the output voltage and power at applied of 30 K were scarcely changed even after ≈1000 cycles of bending and releasing processes with a radius of 5 mm. (Figure 3d). In addition, the Ag_2_Te_0.6_S_0.4_ fiber has excellent long‐term stability (Figure S5a, Supporting Information) and environmental adaptability (Figure S5b, Supporting Information). Thus, appreciable service performance can be expected for this flexible Ag_2_Te_0.6_S_0.4_ fiber.

**Figure 4:**
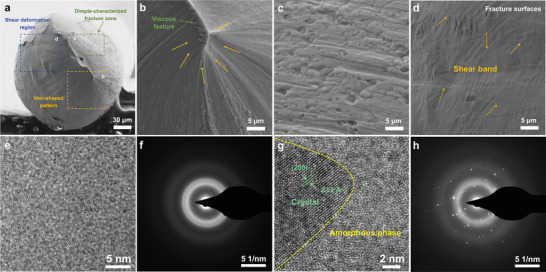
a) Cross‐sectional fracture morphology of Ag_2_Te_0.6_S_0.4_ fiber. b–d) Enlarged images of the rectangles in (a) corresponding to the vein‐shaped region, dimple‐characterized fracture zone and shear deformation region, respectively. e) HRTEM image of pure amorphous phase region and f) the corresponding SAED pattern. g) HRTEM image showing a typical crystallite in the amorphous matrix and h) corresponding SAED pattern.

In order to understand the underlying reason for this brilliant flexibility, the detailed microstructure characterization of Ag_2_Te_0.6_S_0.4_ fiber was performed. After tensile test, fiber shows clear necking and typical cup‐cone plastic characteristics (**Figure** **4**a).^[^ ^39^, ^40^ ^]^ Furthermore, we observe three different fracture morphologies on the fiber fracture surface, which can be mainly divided into the following zones: vein‐shaped region, dimple‐characterized fracture zone and shear deformation region, similar to the previous observed in the bulk sample.^[^ ^31^ ^]^ The typical characteristics of the three zones are shown in Figure 4b‐d, respectively. In the vein‐shaped region (Figure 4b), it can be clearly observed that the vein pattern is spirally distributed with the cone‐shaped viscous characteristic, and become much rougher and deeper along the cone‐shaped center, indicating the high resistance to fracture. Additionally, numerous dimples with sizes ranging from several hundred nanometers to several micrometers are observed in the fracture surface (Figure 4c), similar to many metallic glasses, indicating the plastic flow on the microscale.^[^ ^41^, ^42^ ^]^ The shear deformation region is another typical feature of metallic glasses,^[^ ^43^ ^]^ is observed near the fracture surface and closely connected with the shear band propagation (Figure 4d). Combining the serrations in stress‐strain curves (Figure 3b), which is caused by the multiplication of shear bands,^[^ ^44^ ^]^ it can be deduced that the exception flexibility of Ag_2_Te_0.6_S_0.4_ fiber was associated with the formation and evolution of shear bands. To further understand the microstructure of the TE fiber, high‐resolution transmission electron microscopy (HRTEM) was performed. As illustrated in Figure 4e, the typical structure of amorphous materials without lattice fringes can be observed, and the corresponding selected area electron diffraction (SAED) presents the typical amorphous halo rings (Figure 4f). Additionally, a mixture of amorphous region and lattice stripe region is shown in Figure 4g, where the clearly lattice fringes were observed in this area indicating the high crystalline nature. The measured lattice spacings (2.52 Å) match well with the interplanar spacing of the (200) plane of Ag_2_S_0.7_Te_0.3_ in a body‐centered cubic structure.^[^ ^45^ ^]^ The corresponding SAED pattern is shown in Figure 4h. Both amorphous rings and diffraction spots are found in the SAED pattern, confirming the coexistence of amorphous matrix and crystallites. Similar microstructures are also observed in dendrite metallic glass composites. In this type of materials, nanocrystals act as “soft” elastic/plastic second phase to limit shear band extension, which is generated from the amorphous phase.^[^ ^39^, ^46^ ^]^

### 2.4 TE Performance and Heat Energy Harvesting of TE Textiles

**Figure 5:**
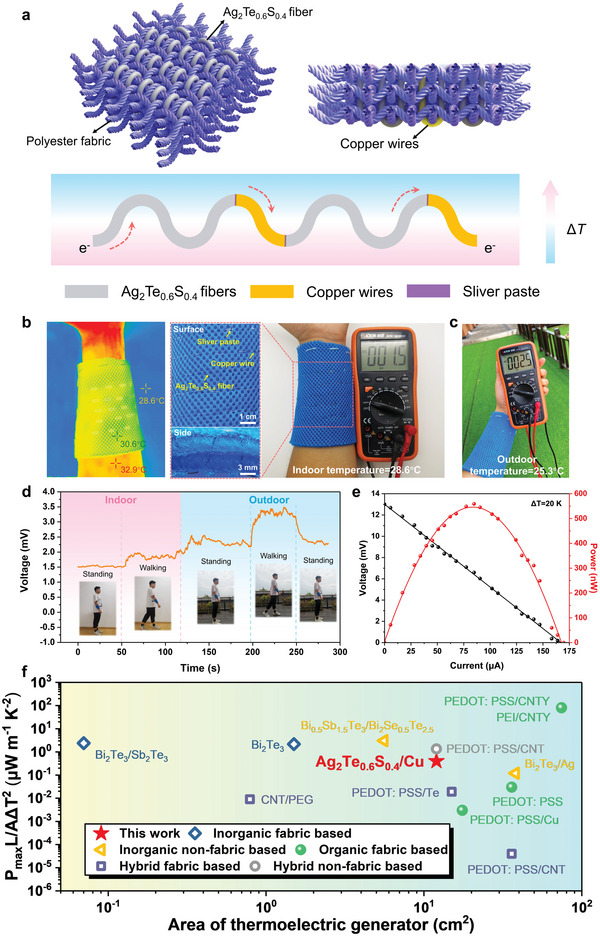
a) A schematic illustration of the configuration of Ag_2_Te_0.6_S_0.4_ fiber series in polyester textile. b) The temperature distribution taken from the infrared camera and the photograph of Ag_2_Te_0.6_S_0.4_ textile for converting human body heat to electric at an ambient temperature 28.6 °C. The insert are surface and side views of the textile. c) The output voltage measured outdoor at 25.3 °C. d) Continuous monitoring on voltage output of the tester wearing the Ag_2_Te_0.6_S_0.4_ textile at standing or walking state indoor or outdoor. e) The output performance of the Ag_2_Te_0.6_S_0.4_ textile at a temperature difference of 20 K. f) The comparison of normalized power density (*P*_max_ *L*/*A*Δ*T* ^2^) of Ag_2_Te_0.6_S_0.4_/Cu TE fabric with the data reported in literature including fabric‐based and nonfabric based TE generators (Table S2, Supporting Information).

The advantageous flexibility, excellent mechanical stability and high TE performance of Ag_2_Te_0.6_S_0.4_ fiber encourage us to investigate its potential application in wearable electronics. As a proof of concept, we fabricated a flexible TE device by weaving Ag_2_Te_0.6_S_0.4_ fibers into a polyester textile, and the detailed structure of the TE textile is shown in **Figure** **5**a. The sixteen Ag_2_Te_0.6_S_0.4_ fibers with the average diameter of 300 µm were woven in the thickness direction of the textile together, which ensures the direct power generation on the human body. To realize the electric connection of all Ag_2_Te_0.6_S_0.4_ fibers in series, copper wires with diameter of 300 µm were wound at one end of each fiber. Consequently, the carriers will flow in the same direction along the fiber, and the voltage multiplication can be achieved. Notably, it is the first time for inorganic semiconductor TE fibers to weave into textile, rather than simply embedding them into clothes. Figure S6a (Supporting Information) also shows the ability of the TE textile to adapt to various loading conditions, such as bending, stretching and folding, and there was essentially no degradation in TE performance due to deformation (Figure S6b,c, Supporting Information).

To evaluate its actual self‐powered characteristics in the human environment, it is fixed directly at the wrist to examine its functions. As shown in Figure 5b, under static circumstance with ambient temperature of 28.6 °C, the temperature difference between the skin temperature (32.9 °C) and surface temperature (30.6 °C) of textile is about 2.3 K, resulting in an output voltage of 1.5 mV. When wearing the TE textile standing in an outdoor environment (25.3 °C), the output voltage is further increased to 2.5 mV, as shown in Figure 5c. The dynamic and continuous monitoring on output performance of TE textile is shown in Figure 5d. Initially, when the volunteer wears TE textile and stands indoors (28.6 °C), the output voltage of the textile is stable at about 1.5 mV. Then, as the volunteer changes from standing still to walking, the output voltage of TE textile increases and remains at a stable voltage level about 1.8 mV. When the volunteer walks from indoor to outdoor and keeps standing, the output voltage quickly increases to 2.5 mV, which is attributed to the larger temperature difference between the skin and textile caused by the lower outdoor temperature. Because of the better heat dissipation under air flow, walking outdoor will further reduce the surface temperature of textile and thus enhance the output voltage to 3.5 mV. When the volunteer stops walking, the output voltage returns to 2.5 mV. It is noteworthy that the temperature difference between the skin and textile is greatly influenced by ambient temperature. For instance, a greater temperature difference (≈20 K) could be achieved in winter or some cold areas on high latitude. In this situation, the TE textile generates the output voltage and the power of 13 mV and 559 nW, respectively (Figure 5e). Besides, as shown in Figure S7 (Supporting Information), the load resistance at the maximum output power of TE textile was ≈79 Ω, which is in accordance with the measured internal resistance of the TE textile (Figure S8, Supporting Information). The subtle deviation should be mainly attributed to the inevitable internal resistance of the multimeter. In Figure 5f, we normalized the reported the TE power outputs and term as maximum normalized power density (*P*_max_ *L*/*A*Δ*T* ^2^, where *P*_max_ is the maximum power output, *L* is the length of fiber, *A* is the cross‐sectional area, and Δ*T* is the temperature difference across the two side of fabric). This normalization is reasonable to evaluate the capability of power generation for all types of wearable TE devices. However, different kinds of TE devices have their own structural configurations and may involve different accessories, it is challenging to assess the device performance in aspect of device size. The Ag_2_Te_0.6_S_0.4_ TE fabric shows a maximum normalized power density as high as ≈0.4 µW m^−1^ K^−2^, which is almost two orders of magnitude higher than the organic TE fabric,^[^ ^47^, ^48^ ^]^ nearly one order of magnitude higher than that of hybrid TE fabric,^[^ ^49^, ^50^ ^]^ and close to inorganic TE fabric.^[^ ^51^, ^52^ ^]^ Higher performance can be expected if the structure of the TE textile is further optimized, such as increase the number of fibers in per unit area and reduce the contact resistance, which would be promising for wearable devices.

## 3 Conclusions

In summary, we have demonstrated a facile molten core method to fabricate inorganic Ag_2_Te_0.6_S_0.4_ TE fibers with both superior flexibility and high TE performance. An extraordinary tensile strain of 21.2% is obtained, which is superior to all the reported inorganic TE fibers and comparable to some typical organic TE fibers. The formation and evolution of shear bands become the unambiguous origin of the exceptional flexibility of Ag_2_Te_0.6_S_0.4_ TE fibers. Benefitted from the unique flexibility, the Ag_2_Te_0.6_S_0.4_ fibers can sustain various complex deformation with excellent long‐term stability and environmental adaptability. As a result, a 3D wearable fabric integrated with Ag_2_Te_0.6_S_0.4_ fibers can be constructed, showing a normalized power density of 0.4 µW m^−1^ K^−2^ under the temperature difference of 20 K, which is approaching to the Bi_2_Te_3_‐based inorganic TE fabric and is almost two orders of magnitude higher than the organic TE fabrics. The simultaneous realization of excellent flexibility and high TE performance establishes the application potential of Ag_2_Te_0.6_S_0.4_ fibers in wearable electronics.

## 4 Experimental Section

### Bulk Materials Synthesis

The Ag_2_Te_0.6_S_0.4_ samples were synthesized by the melting‐annealing method. High‐purity elements, Ag (99.999%, Alfa Aesar), Te (99.99%, Aladdin) and S (99.999%, Aladdin), were weighed with the total mass around 20 g based on the chemical ratio as designed and placed into quartz tube, and then sealed under vacuum of ≈10^−3^ Pa. The quartz tubes were put in the furnace and heated to 1263 K at the rate of 0.3 K min^−1^, homogenized at this temperature for 10 h, and then cooled down to 823 K at the rate of 0.3 K min^−1^. After annealing at this temperature for 3 days, the furnace was cooled down to 393 K and held at this temperature for 10 h. Then, the samples were naturally cooled to room temperature in the furnace. The as‐prepared Ag_2_Te_0.6_S_0.4_ ingots were mechanically grinded to a cylindrical rod with a diameter of 9 mm for the subsequent fiber preparation.

### TE Fiber Fabrication

The TE fiber was fabricated by the molten core method. The obtained Ag_2_Te_0.6_S_0.4_ cylindrical rod with a diameter of 6 mm was inserted into a borosilicate glass tube (glass composition: 73.29SiO_2_·10.09B_2_O_3_·1.25BaO·4.07K_2_O·11.3Na_2_O (molar ratio)) with an inner diameter of 6.1 mm and an external diameter of 20.0 mm to form a preform in the glove box filled with nitrogen. To prevent the volatilization of elements S and Te during the fiber‐drawing process, the opening of the cladding tube was sealed by fireclay. To remove the surface contaminants, both the rod and tube were washed by diluted HCl acid. The preform was suspended in a fiber‐drawing tower and then drawn into fiber at a temperature of 1100 K, which is higher than the melting temperature of Ag_2_Te_0.6_S_0.4_ core. Under this condition, the cladding glass became softened while the Ag_2_Te_0.6_S_0.4_ core is fully melted. The fiber core diameter can be precisely controlled by regulating the drawing speed and preform feed speed.

### Material Characterization

The elemental distribution of fiber section was examined by scanning electron microscopy (SEM, Talos F200x, FEI) equipped with an energy‐dispersive X‐ray spectrometer (EDS). The microstructure of the TE fiber was analyzed by high‐resolution transmission electron microscopy (HR‐TEM, FEI Talos S‐FEG). Typical mechanical measurements under tension for the TE fibers were performed by ZW‐990LA (Dongguan Zhiqu Precision Instrument Co., Ltd). The electrical resistance of as‐prepared fiber was measured by a current–voltage (*I*–*V*) sweeping measurement technique (UNI‐T, UTP1306S). The Seebeck coefficients were investigated by recording the thermoelectric voltage (Δ*V*) induced by a temperature gradient built by thermal resistance. A resistor box (0–9000 Ω) was used to act as an external resistor when testing the output of the Ag_2_Te_0.6_S_0.4_ fiber and TE devices. The detailed calculation of normalized theoretical maximum power density of device is shown in Supporting Note. The temperature profiles of TE device were captured by an infrared camera (IRay, T3S) with the accuracy of ±0.5 °C. The experiments involving human subjects were performed with the full, informed consent of the volunteers, and approval from a national or institutional ethics board/committee was not required.

## Conflict of Interest

The authors declare no conflict of interest.

## Supporting information

Supporting Information

Supplemental Movie 1

Supplemental Movie 2

## Data Availability

The data that support the findings of this study are available from the corresponding author upon reasonable request.
