# Supplementary material for: Superflexible Inorganic Ag2Te0.6S0.4 Fiber with High Thermoelectric Performance
Source: Adv Sci (Weinh). 2023 Mar 8;10(13):2207642. doi: 10.1002/advs.202207642 (PMC10161083; doi:10.1002/advs.202207642)
Supplement: Supplementary file 1 — Supporting Information [file ADVS-10-2207642-s003.pdf]

ADVANCED SCIENCE
Open Access

Supporting Information

for *Adv. Sci.*, DOI 10.1002/advs.202207642

Superflexible Inorganic $Ag_2Te_{0.6}S_{0.4}$ Fiber with High Thermoelectric Performance

Yanqing Fu, Shiliang Kang, Hao Gu, Linling Tan, Chengwei Gao, Zaijin Fang, Shixun Dai and Changgui Lin**

Supporting Information

Superflexible inorganic $Ag_2Te_{0.6}S_{0.4}$ fiber with high thermoelectric performance

Yanqing Fu[1,2,3], Shiliang Kang[1,2,3*], Hao Gu[1,2,3], Linling Tan[1,2,3], Chengwei Gao[1,2,3], Zaijin Fang[4], Shixun Dai[1,2,3], and Changgui Lin[1,2,3*]

[1] *Laboratory of Infrared Materials and Devices, The Research Institute of Advanced Technologies, Ningbo University, Ningbo 315211, P. R. China*

[2] *Key Laboratory of Photoelectric Detection Materials and Devices of Zhejiang Province, Ningbo 315211, P. R. China*

[3] *Engineering Research Center for Advanced Infrared Photoelectric Materials and Devices of Zhejiang Province, Ningbo 315211, P. R. China*

[4] *Guangdong Provincial Key Laboratory of Optical Fiber Sensing and Communications, Institute of Photonics Technology, Jinan University, Guangzhou 511443, China*

***Corresponding author:** kangshiliang@nbu.edu.cn; linchanggui@nbu.edu.cn

This supplement contains

Supporting Figures S1 to S8

Tables S1 to S2

Supporting Movies:

File Name: Supporting Movie S1

Description: The process of $Ag_2Te_{0.6}S_{0.4}$ fiber tied into a knot

File Name: Supporting Movie S2

Description: The stretching process of $Ag_2Te_{0.6}S_{0.4}$ fiber and corresponding stress-strain curve

Supporting Figures

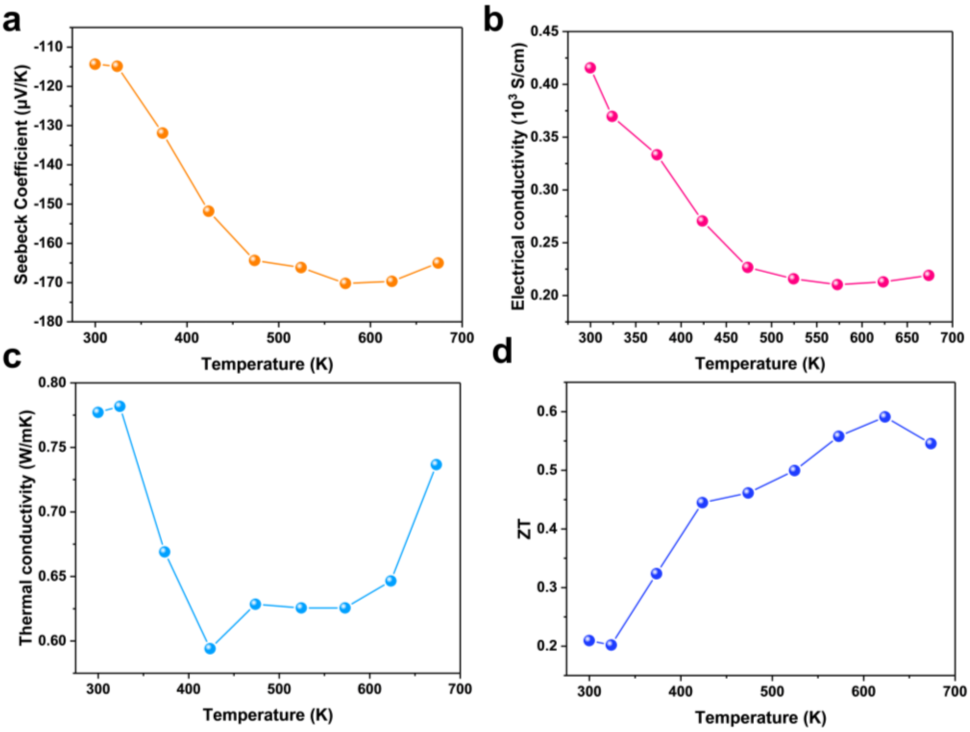

Figure S1. Temperature-dependent thermoelectrical properties of the $Ag_2Te_{0.6}S_{0.4}$ bulk ingots. (a) Seebeck coefficient, (b) Electrical conductivity, (c) total thermal conductivity, and (d) thermoelectrical figure of merit (*ZT*)

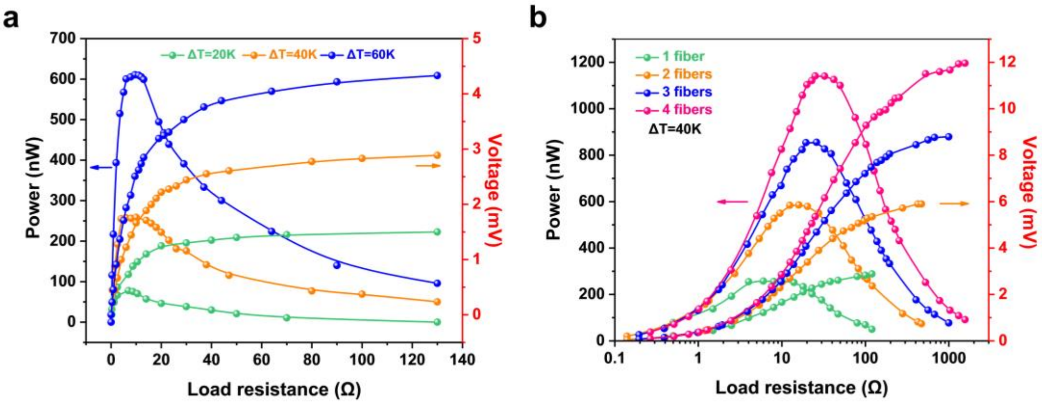

Figure S2. (a) The output voltage and power of one $Ag_2Te_{0.6}S_{0.4}$ fiber as a function of external load resistance at the ΔT ranging from 20 to 60 K. The circuit resistance (R_{total}) at the maximum output power (P_{max}) of a single TE fiber were ≈ 7.5, 7.9, and 8.2 Ω under ΔT of 20, 40, and 60 K, respectively, attributing to the increase of electrical resistivity of $Ag_2Te_{0.6}S_{0.4}$ with increasing temperature. (b) The output voltage

and power as a function of external load resistance under different number of $Ag_2Te_{0.6}S_{0.4}$ fibers in series at a temperature difference of 40 K. The circuit resistance (R_{total}) at the maximum output power (P_{max}) of a single TE fiber were ≈ 7.9, 15.6, 24, and 31.7 Ω.

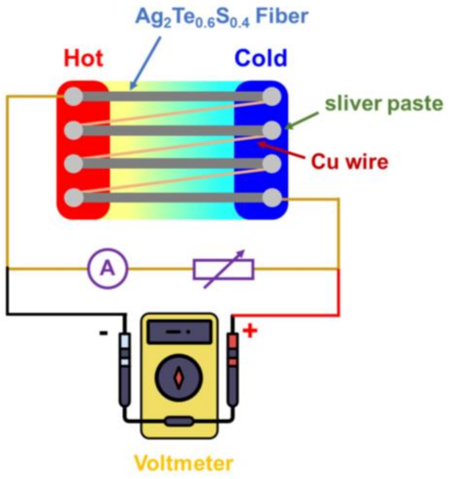

Figure S3. Equivalent electric circuit for measuring fiber output power. The output power is characterized by measured the current through and voltage across external load resistor box which is connected with TE fibers in series.

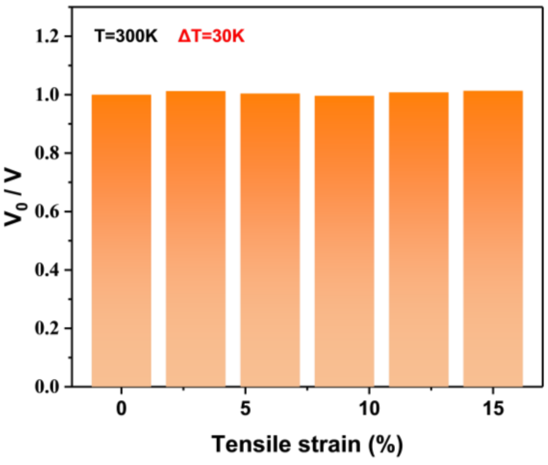

Figure S4. The change of V_0/V of $Ag_2Te_{0.6}S_{0.4}$ fiber under different strains ((V_0/V, where V_0 represents the original output voltage and V is the measured output voltage under deformation)).

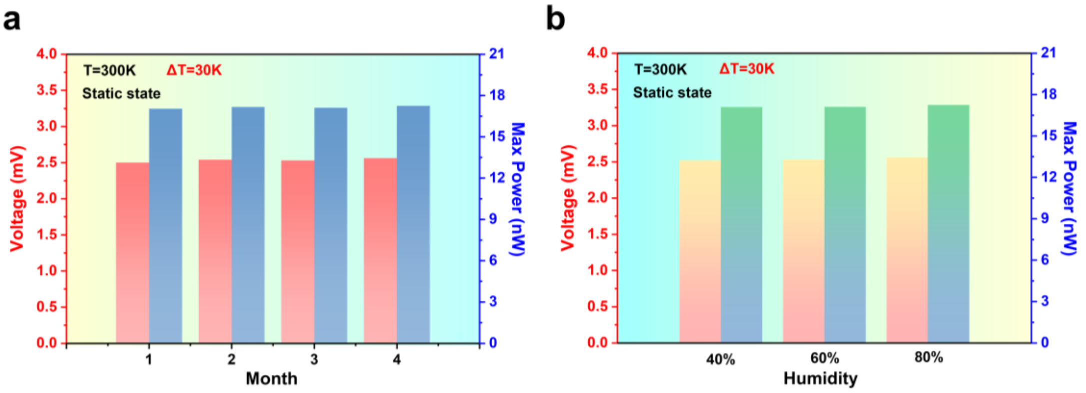

Figure S5. Measured output voltage and max output power for (a) different days and under (b) different humidity (40%, 60%, and 80%). The room temperature is 300 K.

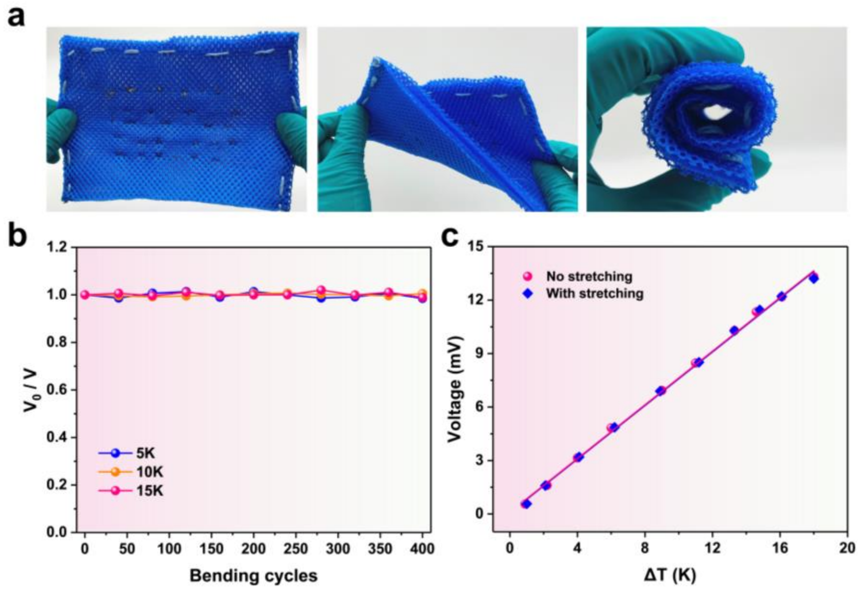

Figure S6. (a) Deformation capacity of TE fabric. (b) Generated voltage from TE fabric as a function of the temperature different with different bending cycles. V_0 is the voltage of TE fabric without bending. (c) Generated voltage from TE fabric as a function of the temperature difference with no stretching and with stretching.

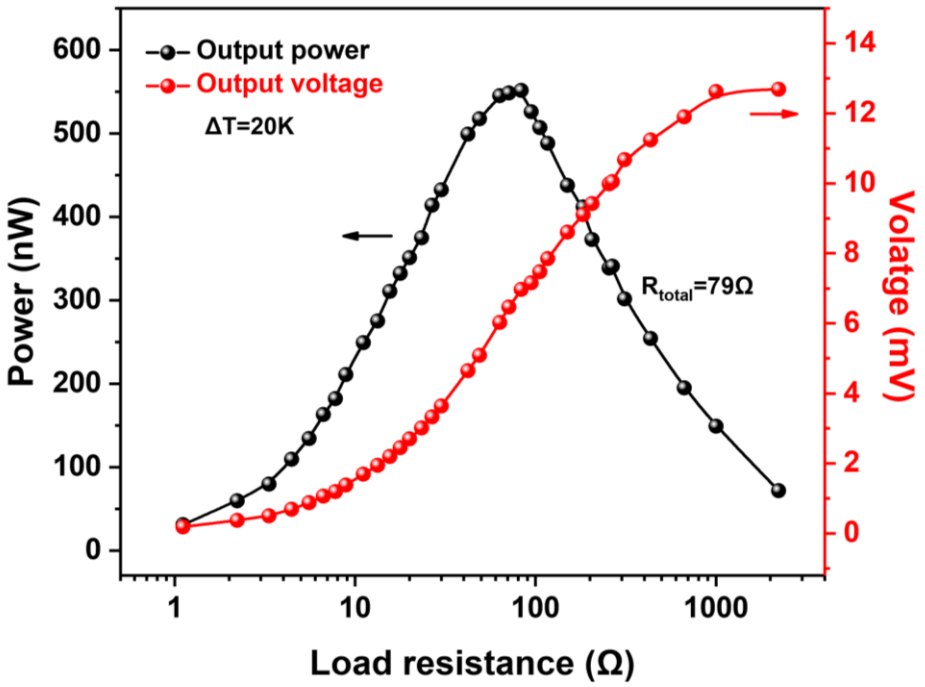

Figure S7. The output voltage and power of the TE textile as a function of external load resistance. It can be seen that the output voltage increases with the external load resistance. The output power increases first and then decreases with the external load resistance, and the maximum output power is ~558 nW, corresponding to the external load resistance of ~79 Ω.

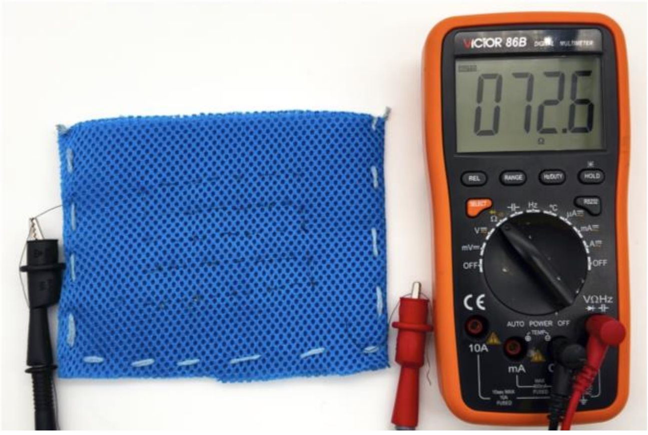

Figure S8. The internal resistance of the $Ag_2Te_{0.6}S_{0.4}$/Cu TE textile.

Supporting Tables

Table. S1 Detailed date and references of TE fibers used in Figure 3a

**Materials**	**Power factor ($\mu Wcm^{-1}K^{-2}$)**	**Maximum tensile strain (%)**	**References**
$Ag_2Te_{0.6}S_{0.4}$	630	21.2	This work
Bi_2Te_3	1300	0.5	[1]
Bi_2Se_3	650	0.3	[2]
$Bi_{0.5}Sb_{1.5}Te_3$	3520	0.3	[2]
Graphene	0.8	1.4	[3]
Polypyrrole/PEDOT: PSS/carbon	45	1.6	[4]
PEDOT:PSS/Te-NWs	28	6.2	[5]
PEDOT: PSS/CNT	10	6.3	[6]
Graphene/PEDOT: PSS	2.9	10.1	[7]
PEDOT: PSS/PVA/Te	3.2	12	[8]
PEDOT: PSS/CNT	40	16	[9]
PEDOT: PSS/Te-NWs	8	17	[10]
PEDOT: PSS/SWCNT	1.8	18	[11]
PEDOT: PSS/CNT	29.3	29	[12]
SWCNT/PVA/PEI	1.2	35	[13]
PEDOT: PSS/DMSO/Ionic Liquid Coating	24	45	[14]
PEDOT: PSS	40	17	[15]
PEDOT: PSS/WPU	25	30	[16]
PEDOT: PSS	160	30.5	[17]
PEDOT: PSS	30	40	[18]

Table. S2 Detailed date and references of TE generators in Figure 5f

**TE fabric type**	**Area of TE fabric (cm^2)**	**ΔT (K)**	**Nos. of TE Legs**	**$P_{max}L/A\Delta T^2$ ($\mu Wm^{-1}K^{-2}$)**	**Heat flow direction**	**References**
Inorganic fabric based	12	20	16	0.4	cross-plane	This work
Inorganic non-fabric based	5.59	35	6	3.06	cross-plane	[19]
Inorganic fabric based	1.5	15	50	2.17	cross-plane	[20]
Inorganic fabric-based	0.07	55	12	2.49	cross-plane	[21]
Inorganic non-fabric based	38	19	20	0.12	in-plane	[1]
Organic fabric based	17.5	74.3	5	0.003	in-plane	[22]
Organic fabric based	36	7.9	36	0.03	cross-plane	[23]
Organic fabric based	36	66	20	0.00004	cross-plane	[24]
Organic fabric based	0.79	5	8	0.009	cross-plane	[25]
Organic fabric based	74.4	47.7	996	80.7	cross-plane	[26]
Hybrid fabric based	15	10	32	0.019	in-plane	[27]
Hybride non-fabric based	12	10	24	1.35	in-plane	[6]

References：

[1] M. Sun, G. Tang, B. Huang, Z. Chen, Y.-J. Zhao, H. Wang, Z. Zhao, D. Chen, Q. Qian, Z. Yang, *J. Materiomics* **2020**, 6, 467.

[2] T. Zhang, K. Li, J. Zhang, M. Chen, Z. Wang, S. Ma, N. Zhang, L. Wei, *Nano Energy* **2017**, 41, 35.

[3] L. Tang, M. He, X. Na, X. Guan, R. Zhang, J. Zhang, J. Gu, *Compos. Commun.* **2019**, 16, 5.

[4] L. Liang, J. Fan, M. Wang, G. Chen, G. Sun, *Compos Sci Technol* **2020**, 187, 107948.

[5] H. Xu, Y. Guo, B. Wu, C. Hou, Q. Zhang, Y. Li, H. Wang, *ACS Appl. Mater. Interfaces* **2020**, 12, 33297.

[6] J.-Y. Kim, W. Lee, Y. H. Kang, S. Y. Cho, K.-S. Jang, *Carbon* **2018**, 133, 293.

[7] J. Liu, G. Liu, J. Xu, C. Liu, W. Zhou, P. Liu, G. Nie, X. Duan, F. Jiang, *ACS Appl. Energy Mater.* **2020**, 3, 6165.

[8] J. Yang, Y. Jia, Y. Liu, P. Liu, Y. Wang, M. Li, F. Jiang, X. Lan, J. Xu, *Compos. Commun.* **2021**, 27, 100855.

[9] C. Xu, S. Yang, P. Li, H. Wang, H. Li, Z. Liu, *Compos. Commun.* **2022**, 32, 101179.

[10] Y. Liu, P. Liu, Q. Jiang, F. Jiang, J. Liu, G. Liu, C. Liu, Y. Du, J. Xu, *Chem. Eng. J.* **2021**, 405, 126510.

[11] J. Liu, Z. Zhu, W. Zhou, P. Liu, P. Liu, G. Liu, J. Xu, Q. Jiang, F. Jiang, *J. Mater. Sci.* **2020**, 55, 8376.

[12] X. Lan, T. Wang, C. Liu, P. Liu, J. Xu, X. Liu, Y. Du, F. Jiang, *Compos Sci Technol* **2019**, 182, 107767.

[13] T. Ding, K. H. Chan, Y. Zhou, X.-Q. Wang, Y. Cheng, T. Li, G. W. Ho, *Nat. Commun.* **2020**, 11, 6006.

[14] M. Li, F. Zeng, M. Luo, X. Qing, W. Wang, Y. Lu, W. Zhong, L. Yang, Q. Liu, Y. Wang, *ACS Appl. Mater. Interfaces* **2021**, 13, 50430.

[15] R. Sarabia-Riquelme, M. Shahi, J. W. Brill, M. C. Weisenberger, *ACS Appl. Polym. Mater.* **2019**, 1, 2157.

[16] N. Wen, Z. Fan, S. Yang, Y. Zhao, C. Li, T. Cong, H. Huang, J. Zhang, X. Guan, L. Pan, *Chem. Eng. J.* **2021**, 426, 130816.

[17] N. Wen, Z. Fan, S. Yang, Y. Zhao, T. Cong, S. Xu, H. Zhang, J. Wang, H. Huang, C. Li, *Nano Energy* **2020**, 78, 105361.

[18] Y. Kim, A. Lund, H. Noh, A. Hofmann, M. Craighero, S. Darabi, S. Zokaei, J. Park, *Macromol Mater Eng* **2020**, 305, 1900749.

[19] Y. Shi, Y. Wang, D. Mei, B. Feng, Z. Chen, *IEEE Robot. Autom. Lett.* **2017**, 3, 373.

[20] M. Hyland, H. Hunter, J. Liu, E. Veety, D. Vashaee, *Appl. Energy.* **2016**, 182, 518.

[21] J. A. Lee, A. E. Aliev, J. S. Bykova, M. J. de Andrade, D. Kim, H. J. Sim, X. Lepró, A. A. Zakhidov, J. B. Lee, G. M. Spinks, *Adv. Mater.* **2016**, 28, 5038.

[22] Y. Du, K. Cai, S. Z. Shen, R. Donelsonand, J. Xu, H. Wang, T. Lin, *RSC Adv.* **2017**, 7, 43737.

[23] T. Park, H. Lim, J. U. Hwang, J. Na, H. Lee, E. Kim, *APL Mater.* **2017**, 5, 074106.

[24] Q. Wu, J. Hu, *Smart Mater Struct* **2017**, 26, 045037.

[25] M. Ito, T. Koizumi, H. Kojima, T. Saito, M. Nakamura, *J. Mater. Chem. A* **2017**, 5, 12068.

[26] Y. Zheng, Q. Zhang, W. Jin, Y. Jing, X. Chen, X. Han, Q. Bao, Y. Liu, X. Wang, S. Wang, *J. Mater. Chem. A* **2020**, 8, 2984.

[27] E. Jin Bae, Y. Hun Kang, K.-S. Jang, S. Yun Cho, *Sci. Rep.* **2016**, 6, 1.
